# CO_2_ modulation of the rates of photosynthesis and light-dependent O_2_ consumption in *Trichodesmium*

**DOI:** 10.1093/jxb/ery368

**Published:** 2018-10-30

**Authors:** Tobias G Boatman, Phillip A Davey, Tracy Lawson, Richard J Geider

**Affiliations:** 1 School of Biological Sciences, University of Essex, Wivenhoe Park, Colchester, UK; 2 Department of Chemical Engineering, Imperial College London, South Kensington, London, UK

**Keywords:** Carbon fixation, CO_2_, cyanobacteria, gross photosynthesis, net photosynthesis, ocean acidification, *Trichodesmium*

## Abstract

As atmospheric CO_2_ concentrations increase, so too does the dissolved CO_2_ and HCO_3_^–^ concentrations in the world’s oceans. There are still many uncertainties regarding the biological response of key groups of organisms to these changing conditions, which is crucial for predicting future species distributions, primary productivity rates, and biogeochemical cycling. In this study, we established the relationship between gross photosynthetic O_2_ evolution and light-dependent O_2_ consumption in *Trichodesmium erythraeum* IMS101 acclimated to three targeted pCO_2_ concentrations (180 µmol mol^–1^=low-CO_2_, 380 µmol mol^–1^=mid-CO_2_, and 720 µmol mol^–1^=high-CO_2_). We found that biomass- (carbon) specific, light-saturated maximum net O_2_ evolution rates (P_nC,max_) and acclimated growth rates increased from low- to mid-CO_2_, but did not differ significantly between mid- and high-CO_2_. Dark respiration rates were five times higher than required to maintain cellular metabolism, suggesting that respiration provides a substantial proportion of the ATP and reductant for N_2_ fixation. Oxygen uptake increased linearly with gross O_2_ evolution across light intensities ranging from darkness to 1100 µmol photons m^–2^ s^–1^. The slope of this relationship decreased with increasing CO_2_, which we attribute to the increased energetic cost of operating the carbon-concentrating mechanism at lower CO_2_ concentrations. Our results indicate that net photosynthesis and growth of *T. erythraeum* IMS101 would have been severely CO_2_ limited at the last glacial maximum, but that the direct effect of future increases of CO_2_ may only cause marginal increases in growth.

## Teaser

The ratio of light-driven O_2_ consumption to gross photosynthetic O_2_ evolution decreased significantly with increasing CO_2_, which is probably due to a reduced cost of operating the carbon-concentrating mechanism.

## Introduction

The ocean is one of the largest readily exchangeable reservoirs of inorganic carbon on Earth and is a major sink for anthropogenic CO_2_ emissions (Sabine *et al.*, 2004). The ocean’s capacity to sequester atmospheric CO_2_ is strongly mediated by biological processes (Raven and Falkowski, 1999), where organic matter production and export drive CO_2_ sequestration. This is important as future emission scenarios predict that atmospheric CO_2_ will increase from present concentrations (~400 µmol mol^–1^) to 750 µmol mol^–1^ or 1000 µmol mol^–1^ by the end of this century (Raven *et al.*, 2005). This will lead to an increase in the total dissolved inorganic carbon (TIC) in the surface ocean, reducing the pH from an average value of ~8.2 (pre-industrial) to ~7.9 (estimated for 2100) (Zeebe *et al.*, 1999; Zeebe and Wolf-Gladrow, 2001). Ocean acidification therefore favours an increase in seawater CO_2_ and HCO_3_^–^ concentration and a decrease in pH and CO_3_^2–^.

There are still many uncertainties regarding the biological response of key groups of organisms to these changing conditions, which is crucial for predicting future species distributions, primary productivity rates, and biogeochemical cycling. One group of great importance are diazotrophic cyanobacteria (photosynthetic dinitrogen fixers), as they contribute significantly to overall marine primary productivity by providing new nitrogen to many oligotrophic areas of the oceans. The filamentous cyanobacteria *Trichodesmium* are a colony-forming species that forms extensive surface blooms in the tropical and subtropical oceans (Carpenter and Capone, 1992; Capone *et al.*, 1997; Campbell *et al.*, 2005). *Trichodesmium* plays a significant role in the N cycle of the oligotrophic oceans; fixing nitrogen in an area corresponding to half of the Earth’s surface (Davis and McGillicuddy, 2006) and representing up to 50% of new production in some oligotrophic tropical and subtropical oceans (Capone, 2005). The annual marine N_2_ fixation is currently estimated at between 100 Tg and 200 Tg N per year (Gruber and Sarmiento, 1997; Karl *et al.*, 2002), of which *Trichodesmium* spp. contribute between 80 Tg and 110 Tg of fixed N_2_ to open ocean ecosystems (Capone *et al.*, 1997).

Cyanobacteria have performed oxygenic photosynthesis for ~2.7 billion years (Buick, 2008). During that time, CO_2_ concentrations have declined and O_2_ concentrations increased, thus exerting an evolutionary pressure to form a mechanism to reduce the impact of photorespiration on photosynthetic CO_2_ fixation. Despite cyanobacterial Rubisco having a relatively low affinity for CO_2_, cyanobacteria achieve high photosynthetic rates by virtue of an intracellular carbon-concentrating mechanism (CCM), which thereby reduces the diversion of energy into oxygenation of ribulose-1,5-bisphosphate (RuBP), the first step in photorespiration (Schwarz *et al.*, 1995; Kaplan and Reinhold, 1999). In addition, the CCM can aid in the dissipation of excess light energy as well as maintaining an optimal intracellular pH (Badger *et al.*, 1994; Kaplan and Reinhold, 1999).

Cyanobacteria have a unique ability to perform both photosynthesis and respiration simultaneously in the same cellular compartment (Nagarajan and Pakrasi, 2001). The thylakoid membranes of cyanobacteria contain both respiratory and photosynthetic electron transport chains, sharing the plastoquinone and plastocyanin pools and the Cyt *b*_6_*f* complex. In contrast, the cytoplasmic membrane is only capable of performing respiratory electron transport (Nagarajan and Pakrasi, 2001). Thus, it is common in cyanobacteria for respiratory electron transport to be inhibited at low light intensities as photosynthesis increases in the thylakoid membranes (Kana, 1992). However, in *Trichodesmium* there remains the possibility that photosynthetic and respiratory metabolism differs between diazocytes (where N_2_ fixation occurs) and other cells within a trichome.

Previous studies report an increase in growth and productivity (CO_2_ and N_2_ fixation) of *T. erythraeum* IMS101 as well as changing elemental composition in response to future CO_2_ concentrations (~750–1000 µmol mol^–1^) (Barcelos e Ramos *et al.*, 2007; Levitan *et al.*, 2007, 2010*a*; Kranz *et al.*, 2010; Spungin *et al.*, 2014; Hutchins *et al.*, 2015; Boatman *et al.*, 2017, 2018*a, b*), although, as discussed in Boatman *et al.* (2018*a*), the magnitude of the responses often differs between studies. Due to the significant contribution that *Trichodesmium* makes to biogeochemical cycles and the predicted change in inorganic carbon (Ci) speciation over the coming decades, we performed a systematic experiment to assess how the photosynthetic physiology of *T. erythraeum* IMS101 was affected by acclimation to varying CO_2_. We ensured that the Ci chemistry and all other growth conditions were well defined, with cultures fully acclimated over long time periods (~5 months) to achieve balanced growth. We assessed the dark respiration, light absorption, and the light dependencies of gross O_2_ evolution and O_2_ consumption across different CO_2_ conditions. We discuss how the responses that we observed may be related to N_2_ fixation and changes in the cost of operating the CCM.

## Materials and methods

*Trichodesmium erythraeum* IMS101 was semi-continuously cultured to achieve fully acclimated balanced growth at three target pCO_2_ concentrations (180, 380, and 720 µmol mol^–1^), under saturating light intensity (400 µmol photons m^–2^ s^–1^), a 12/12 h light/dark (L/D) cycle, and an optimum growth temperature (26 ± 0.7 °C) for ~5 months (~40, 70, and 80 generations at low-, mid-, and high-CO_2_, respectively).

### Experimental set-up

Cultures of *T. erythraeum* IMS101 were grown in standard YBCII medium (Chen *et al.*, 1996) under diazotrophic conditions (N_2_ only) in 1.5 litre volumes in 2 litre Pyrex bottles that had been acid-washed and autoclaved prior to culturing. Illumination was provided side-on by fluorescent tubes (Sylvania Luxline Plus FHQ49/T5/840). Cultures were constantly mixed using magnetic PTFE stirrer bars and aerated with a filtered (0.2 µm pore) air mixture at a rate of ~200 ml s^–1^. The CO_2_ concentration was regulated (±2 µmol mol^–1^) by mass-flow controllers (Bronkhorst, Newmarket, UK) and CO_2_-free air was supplied by an oil-free compressor (Bambi Air, UK) via a soda-lime gas-tight column that was mixed with a 10% CO_2_-in-air mixture from a gas cylinder (BOC Industrial Gases, UK). The CO_2_ concentration in the gas phase was continuously monitored by an infra-red gas analyser (Li-Cor Li-820, Lincoln, NE, USA), calibrated weekly against a standard gas (BOC Industrial Gases).

Cultures were kept at the upper section of the exponential growth phase through periodic dilution with new growth media at 3–5 d intervals. Daily growth rates were quantified from changes in baseline fluorescence (*F*_o_) measured between 09.00 h and 10.30 h on dark-adapted cultures (20 min) using a FRRfII FastAct Fluorometer System (Chelsea Technologies Group Ltd, UK). As detailed in Boatman *et al.* (2018*a*), cultures were deemed fully acclimated and in balanced growth when both the slope of the linear regression of ln(*F*_o_) and the ratio of live-cell to acetone-extracted *F*_o_ were constant following every dilution with fresh YBCII medium.

The Ci chemistry was measured prior to the dilution of each culture with fresh media, where exactly 20 ml of culture from each treatment was filtered through a swinnex filter (25 mm, 0.45 µm pore, glass fibre filter): 15 ml into a plastic centrifuge tube (no headspace) for TIC analysis (Shimadzu TOC-V Analyser & ASI-V Autosampler), and 5 ml into a plastic cryogenic vial (Sigma-Aldrich V5257-250EA; no headspace) for pH analysis.The bicarbonate (HCO_3_^–^), carbonate (CO_3_^2–^), and CO_2_ concentrations were calculated via *CO2SYS* as described in Boatman *et al.* (2017). Overall, the CO_2_ drawdown in the cultures ranged between 49 µmol mol^–1^ and 90 µmol mol^–1^ for all CO_2_ treatments (Table 1) and exhibited a negligible CO_2_ drift over a diurnal cycle (see Supplementary Fig. S1 at *JXB* online).

**Table 1.** The growth conditions (±SE) achieved for *T. erythraeum* IMS101 when cultured at three target gas phase ChlCO_2_ concentrations (low=180 µmol mol^–1^, mid=380 µmol mol^–1^, and high=720 µmol mol^–1^), saturating light intensity (400 µmol photons m^–2^ s^–1^), and optimal temperature (26 °C)

Variables	Units	Low-CO_2_	Mid-CO_2_	High-CO_2_
pH	–	8.461	8.175	7.905
H^+^	nM	3.5 (0.1)	6.7 (0.1)	12.5 (0.2)
A_T_	µM	2427 (32)	2490 (51)	2444 (42)
TCO_2_	µM	1797 (30)	2076 (44)	2204 (37)
HCO_3_^–^	µM	1356 (30)	1773 (37)	2008 (32)
CO_3_^2–^	µM	436 (9)	295 (8)	179 (5)
CO_2_	µM	3.3 (0.2)	8.2 (0.2)	17.4 (0.3)
NH_4_^+^	mM	1.00 (0.12)	1.06 (0.08)	1.02 (0.06)
NO_3_^–^	mM	0.33 (0.05)	0.36 (0.02)	0.32 (0.02)
*n*		76	32	28

Individual pH values were converted to a H^+^ concentration, allowing a mean pH value to be calculated. Dissolved inorganic NH_4_^+^ was determined using the phenol-hypochlorite method as described by Solorzano (1969), while dissolved inorganic NO_3_^–^ was determined using the spectrophotometric method as described by Collos *et al.* (1999).

### Gross and net O_2_ exchange

Light-dependent rates of O_2_ production and consumption were measured on four biological replicates per CO_2_ treatment, using a membrane inlet mass spectrometer (MIMS) and an ^18^O_2_ technique modified from McKew *et al.* (2013).

MIMS samples were prepared by placing 300 ml of culture in a large gas-tight syringe, and gently bubbled with N_2_ gas for ~20 min to reduce the ^16^O_2_ concentration. The headspace was removed, and 2 ml of ^18^O_2_ gas (CK Gas Products, UK; 99% purity) was added and mixed by continuously inverting the syringe for 20 min. During this process, the culture was maintained at a low light intensity (<10 µmol photons m^–2^ s^–1^) and at growth temperature (26 °C). Samples were incubated using a series of 6 ml glass stopper, gas-tight test tubes, which were cleaned with detergent, acid-washed (10% HCl for 1 d), and rinsed with deionized water (Millipore Milli-Q Biocel, ZMQS60FOI) prior to use. Glass beads were placed inside each test tube, allowing the sample to be mixed throughout the incubation. The ^18^O_2_-enriched culture was quickly dispensed into the gas-tight glass test tubes, sealed using ground glass stoppers (no headspace), and immediately placed into a temperature-controlled (26 °C) incubator. A white light-emitting diode (LED) block (Iso Light 400, Technologica, Essex, UK) was positioned at one end of the incubator, generating light intensities ranging from 10 µmol photons m^–2^ s^–1^ to 1100 µmol photons m^–2^ s^–1^.

For each replicate, 24 test tubes were incubated across the light gradient, a minimum of 10 test tubes were used to determine the initial concentration of O_2_ isotopes, and an additional four test tubes were incubated in the dark (26 °C) to determine dark respiration rates. All photosynthesis–light (P–E) response curves were measured at the same time of day between 4 h and 6 h into the photo-phase of the L/D cycle and were incubated for between 60 min and 120 min. Culture densities for these experiments ranged from 80 µg Chl *a* l^–1^ to 240 µg Chl *a* l^–1^.

Changes in ^16^O_2_ and ^18^O_2_ and thus O_2_ consumption (U_0_) and O_2_ evolution (E_0_) were calculated using the following equations (Radmer and Kok, 1976);

U0=−(1+O 162O 182) ⋅ ΔO 182Δt (1)

E0=ΔO 162Δt−(O 162O 182) ⋅ ΔO 182Δt (2)

where U_0_ is the rate of O_2_ consumption and E_0_ is the rate of gross O_2_ evolution. C-specific rates were obtained by dividing U_0_ and E_0_ by the concentration of particulate organic carbon (POC). Rates were also normalized to Chl *a* and particulate organic nitrogen (PON), and are presented in Supplementary Figs S2 and S3.

The P–E curves for gross (E_0C_) and net O_2_ exchange (P_nC_=E_0C_–U_0C_) were fitted to the following equations from Platt and Jassby (1976);

$${\text{E}}_{0\text{C}}={\text{E}}_{0\text{C},\text{max}}\cdot \;\left[1-\text{e}\left(\frac{-\alpha_{\text{gC}}\;\cdot \text{ E}}{{\text{E}}_{0\text{C},\text{max}}}\right)\right]$$ (3)

$${\text{P}}_{\text{nC}}={\text{ P}}_{\text{nC},\text{max}}\;\cdot \;\left[1-\text{e}\left(\frac{-\alpha_{\text{nC}}\;\cdot \text{ E}}{{\text{P}}_{\text{nC},\text{max}}}\right)\right]+{\text{ R}}_{\text{dC}}$$ (4)

where E_0C,max_ and P_nC,max_ are the carbon-specific maximum gross and net O_2_ evolution rates; α_gC_ and α_nC_ are the carbon-specific initial light-limited slopes for gross and net photosynthesis; R_dC_ is the dark respiration rate; and E is the light intensity (µmol photons m^–2^ s^–1^). Curve fitting was performed on each biological replicate separately to calculate mean (±SE) curve fit parameterizations (Sigmaplot 11.0).

The maximum quantum efficiencies of gross (ϕ_m,g_) and net (ϕ_m,n_) O_2_ evolution were calculated as follows;

$$\phi_{\text{m},\text{g}}=\frac{\alpha_{\text{gC}}}{{\text{a}}_{\text{C},\text{eff}}}$$ (5)

$$\phi_{\text{m},\text{n}}=\frac{\alpha_{\text{nC}}}{{\text{a}}_{\text{C},\text{eff}}}$$ (6)

where the C-specific initial slope for gross (α_gC_) or net (α_nC_) O_2_ evolution, spectrally corrected to the culturing LEDs (Supplementary Fig. S6), was divided by the C-specific, spectrally corrected effective light absorption coefficient (a_C,eff_).

### Spectrophotometric Chl *a* and POC analysis

Samples for the determination of Chl *a* and POC were collected with each light–response curve, while PON was calculated from the measured POC using the CO_2_-specific C:N ratio reported in Boatman *et al.* (2018*a*). For measurements of Chl *a* and POC, two 100 ml samples from each culture were vacuum-filtered onto pre-combusted 25 mm glass fibre filters (0.45 µm pore; Fisherbrand FB59451, UK). The first filter was dried at 60 °C and the POC quantified using a TC analyser (Shimadzu TOC-V Analyser & SSM-5000A Solid Sample Combustion Unit). The second filter was placed in 5 ml of 100% methanol, homogenized, and extracted overnight at –20 °C, before being centrifuged at 12 000 *g* for 10 min, and a 3 ml aliquot of the supernatant added to a quartz cuvette. The absorption spectrum (400–800 nm) was measured using a (Hitachi U-3000, Japan) spectrophotometer and the Chl *a* concentration (µg l^–1^) was calculated using the following equation (Ritchie, 2008);

$$\text{Chl}\;a\;=\left[\frac{\left(12.9447\;\cdot \;\left(Abs_{665}\;-\;Abs_{750}\right)\right)\;\cdot \;Vol_{E}}{Vol_{F}}\right]\cdot 1000$$ (7)

where Abs_665_ and Abs_750_ are the baseline-corrected optical densities of the methanol-extracted sample at 665 nm and 750 nm; Vol_E_ is the volume of the solvent used for extraction (i.e. 5 ml); Vol_F_ is the volume of culture filtered (i.e. 100 ml), and 12.9447 is a cyanobacteria-specific Chl *a* coefficient for 100% methanol extraction.

Supporting spectrophotometric measurements were made on live cells using an integrating sphere to determine the *in vivo* light absorption (Supplementary File SI). From this we determined biomass-specific (Chl *a*, C, and N) light absorption coefficients under the varying CO_2_ treatments (Supplementary Fig. S4), reconstructed the light absorption spectra from photosynthetic pigment spectra (Supplementary File SII; Supplementary Table S1), and calculated maximum quantum efficiencies of gross and net O_2_ evolution (Table 3).

## Results

### Growth rate, cell composition, and light absorption

Balanced growth rates increased from 0.2 d^–1^ at low-CO_2_ to 0.34 d^–1^ at mid-CO_2_ and 0.36 d^–1^ at high-CO_2_ (Table 2). Chl *a*:C ratios were lowest under low-CO_2_ conditions and were significantly higher in the mid-CO_2_ treatment relative to the low- and high-CO_2_ treatments (Table 2).

**Table 2.** The mean (±SE) balanced growth rate and Chl *a*:C ratio for *T. erythraeum* IMS101 when acclimated to three target CO_2_ concentrations (low=180 µmol mol^–1^, mid=380 µmol mol^–1^, and high=720 µmol mol^–1^), saturating light intensity (400 µmol photons m^–2^ s^–1^), and optimal temperature (26 °C)

Variables	Units	Low-CO_2_	Mid-CO_2_	High-CO_2_
Growth rate	d^–1^	0.198 (0.027) A	0.336 (0.026) B	0.361 (0.020) B
Chl *a*:C	g:mol	0.052 (0.003) A	0.089 (0.003) C	0.066 (0.003) B

Abbreviations: Chl *a*:C ratios are g:mol (*n*=9 at low-CO_2_, *n*=6 at mid- and high-CO_2_). Letters indicate significant differences between CO_2_ treatments (one-way ANOVA, Tukey post-hoc test; *P*<0.05); where B is significantly greater than A, and C is significantly greater than B and A.

### Light dependence of O_2_ exchange

The C-specific maximum rate (E_0C,max_) and initial slope (α_gC_) of light-dependent gross photosynthesis were significantly higher in the mid-CO_2_ treatment relative to the low- and high-CO_2_ treatments (Table 3). Conversely, the light saturation parameter (E_k_=E_0C,max_/α_gC_) for gross O_2_ evolution (Table 3) and the maximum quantum efficiency of gross O_2_ evolution (ϕ_m,g_=α_gC_/a_C,eff_) (Table 3) did not vary significantly amongst the CO_2_ treatments due to co-variation of α_gC_ and E_0C,max_.

**Table 3.** The physiological parameters (±SE) of the C-specific light–response curves for the gross and net photosynthetic O_2_ evolution of *T. erythraeum* IMS101 (*n*=4) measured using the MIMS light source

Parameters	Units	Low-CO_2_	Mid-CO_2_	High-CO_2_
Gross O_2_ evolution				
E_0C,max_	mmol O_2_ (g C)^–1^ h^–1^	1.875 (0.118) A	3.795 (0.175) C	2.973 (0.158) B
E_k_	µmol photons m^-2^ s^–1^	277 (15)	250 (20)	281 (15)
α_gC_	µmol O_2_ (g C)^–1^ h^–1^ (µmol photons m^-2^ s^-1^)^–1^	6.78 (0.33) A	15.35 (0.82) C	10.58 (0.18) B
ϕ_m,g_	mol O_2_ (mol photons)^–1^	0.037 (0.004)	0.042 (0.004)	0.045 (0.003)
Net photosynthesis				
P_nC,max_	mmol O_2_ (g C)^–1^ h^–1^	1.131 (0.061) A	2.534 (0.287) B	2.312 (0.140) B
E_k_	µmol photons m^–2^ s^–1^	300 (41)	270 (24)	270 (10)
α_nC_	µmol O_2_ (g C)^–1^ h^–1^ (µmol photons m^–2^ s^–1^)^–1^	3.94 (0.46) A	9.48 (1.02) B	8.57 (0.35) B
R_dC_	mmol O_2_ (g C)^–1^ h^–1^	–0.600 (0.078)	–0.644 (0.132)	–0.659 (0.013)
ϕ_m,n_	mol O_2_ (mol photons)^–1^	0.020 (0.002) A	0.026 (0.003) A	0.037 (0.002) B
Slopes				
P_nC_ versus E_0C_	Dimensionless	0.571 (0.028) A	0.646 (0.046) A	0.791 (0.017) B
U_0C_ versus E_0C_	Dimensionless	0.429 (0.028) B	0.354 (0.046) A	0.209 (0.017) A
U_0C_ versus P_nC_	Dimensionless	0.701 (0.073) B	0.553 (0.111) B	0.254 (0.024) A

Abbreviations: E_0C,max_, the C-specific maximum gross O_2_ evolution rate; P_nC,max_, the C-specific maximum net O_2_ evolution rate; E_k_, the light saturation parameter; α_gC_ and α_nC_ are the C-specific initial slopes of the light–response curve for net and gross photosynthesis; ϕ_m,g_ and ϕ_m,n_ are the maximum quantum efficiencies of gross and net O_2_ evolution calculated using the absorption coefficients reported in Supplementary Table S1; R_dC_, the C-specific dark respiration rate; Slope=the slope of the regression of E_0C_ against P_nC_, E_0C_ against O_2_ uptake (U_0C_), and U_0C_ against P_nC_. Letters indicate significant differences between CO_2_ treatments (one-way ANOVA, Tukey post-hoc test; *P*<0.05); where B is significantly greater than[A, and C is significantly greater than B and A.

The C:N ratios reported by Boatman *et al.* (2018*a*) for the low-, mid-, and high-CO_2_ treatments (7.9, 7.8, and 7.3 mol:mol, respectively) were not significantly different. As such, the CO_2_ response of N-specific maximum rates and light-limited initial slopes of gross O_2_ evolution were comparable with C-specific rates (Supplementary Fig. S2; Supplementary Table S2). The ~2-fold variability of E_0C,max_ and α_gC_ was largely due to differences in the Chl *a*:C ratio, with the Chl *a*-specific light absorption varying by only 1% across CO_2_ treatments (Supplementary Table S1).

The C-specific dark respiration rates (R_dC_) varied by ~10% amongst CO_2_ treatments (Table 3). The maximum net O_2_ evolution rate (P_nC,max_) approximately doubled from the low-CO_2_ to the mid- and high-CO_2_ treatments, but did not differ between mid- and high-CO_2_, with the initial slope (α_nC_) showing a similar pattern to P_nC,max_ (Table 3). Similar responses were observed for the maximum rate (V_C,max_) and initial slope (Affinity) of the CO_2_ dependency of C fixation as reported in Boatman *et al.* (2018*a*).

**Fig. 1.**
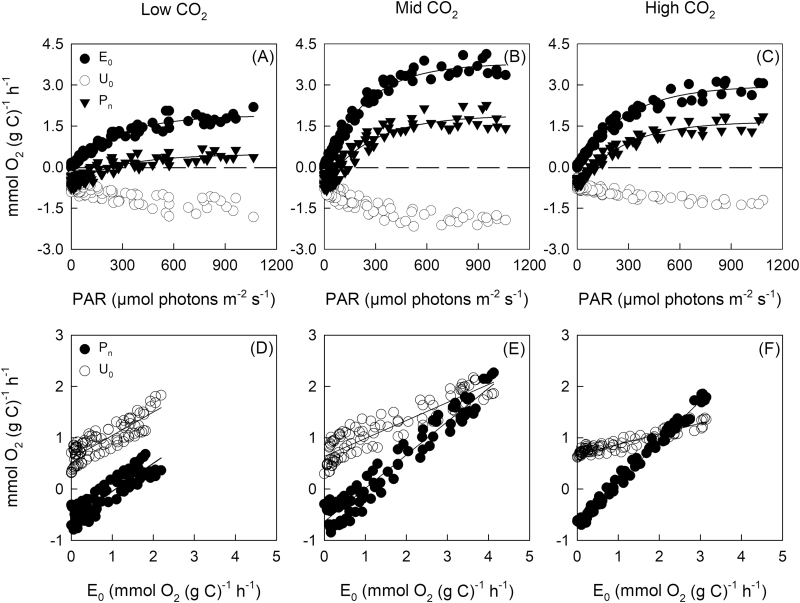
The C-specific light–response curves for gross O_2_ evolution, O_2_ consumption, and net photosynthesis (*n*=4) (A–C) and the relationship between gross net O_2_ evolution and net O_2_ evolution or O_2_ consumption (D–F) for *T. erythraeum* IMS101. Oxygen evolution rates are normalized to a carbon basis [mmol O_2_ (g C)^–1^ h^–1^]. The dashed line represents where gross O_2_ evolution equals O_2_ consumption (i.e. net photosynthesis=0). Note, Chl *a*- and N-specific light–response curves are reported in Supplementary Figs S2 and S3.

The relationship between P_n_ and E_0_ was linear (Fig. 1), with the slope increasing by ~13% from low- to mid-CO_2_ and by 22% from mid- to high-CO_2_ (Table 4). This linear relationship indicates that light-dependent O_2_ consumption (U_0C_) was a constant proportion of gross O_2_ evolution (E_0C_), independent of light intensity under each of the CO_2_ treatments. Subtracting the slope from unity gives the ratio of light-dependent O_2_ consumption to gross O_2_ evolution, which declined significantly from 0.79 at high-CO_2_ to 0.65 at mid-CO_2_ and 0.57 at low-CO_2_.

**Table 4.** The photosynthetic quotients (±SE) for *T. erythraeum* IMS101, calculated from the light-saturated, maximal rates of carbon-specific O_2_ evolution, and the C fixation rates

Parameters	Units	Low-CO_2_	Mid-CO_2_	High-CO_2_
Photosynthetic quotient				
E_0C_/V_C_	mol O_2_ (mol C)^–1^	1.98 (0.10)	1.99 (0.04)	1.28 (0.04)
P_nC_/V_C_	mol O_2_ (mol C)^–1^	1.14 (0.07)	1.29 (0.11)	1.01 (0.05)

V_C_ was calculated as V_C_=(V_C,max_×[CO_2_])/(K_m_+[CO_2_]) using the value of K_m_ from Boatman *et al.* (2018*a*) and [CO_2_] from Table 1. E_0C_ was calculated as E_0C_=E_0C,max_×[1–e(–α×E/E_0C,max_)] and P_nC_ was calculated as P_nC_=P_nC,max_×[1–e(–α×E/P_nC,max_)] using E=400 µmol photons m^–2^ s^–1^ and values of E_k_ from Table 3.

### Photosynthetic quotient

We calculated the photosynthetic quotient (*PQ*) as:

$$PQ=\frac{\left({\text{P}}_{\text{nC}}\right)}{{\text{V}}_{C}}$$ (8)

where P_nC_ is the C-specific net O_2_ evolution rate (E_0_–U_0_) and V_C_ is the C-specific C-fixation rate reported by Boatman *et al.* (2018*a*), with both P_nC_ and V_C_ calculated at the growth light intensity (400 mmol photon m^–2^ s^–1^) and growth CO_2_ concentration (Table 1). The *PQ* calculated in this way (Table 4) did not vary systematically amongst the CO_2_ treatments, averaging about 1.15 mol O_2_ mol CO_2_^–1^.

A second value of *PQ* was calculated by dividing E_0C_ by V_C_ for C fixation under the corresponding conditions, which increased from 1.3 mol O_2_ mol CO_2_^–1^ in the high-CO_2_ treatment to ~2.0 mol O_2_ mol CO_2_^–1^ for the low- and mid-CO_2_^1^ treatments (Table 4), reflecting the increase in light-dependent O_2_ consumption with decreasing CO_2_.

## Discussion

### Effect of acclimation to variation of inorganic chemistry on growth rates and the Chl *a*:carbon ratio

The increased growth rate from low- (180 µmol mol^–1^) to mid- (380 µmol mol^–1^) and high-CO_2_ (720 µmol mol^–1^) was similar to previous findings (Barcelos e Ramos *et al.*, 2007; Boatman *et al.*, 2017, 2018*b*). Whilst not statistically significant, balanced growth rates were ~10% greater at high-CO_2_ than at mid-CO_2_. The magnitude of this increase is comparable with several recent studies, which show rates increasing by 7–26% at similar CO_2_ concentrations (Barcelos e Ramos *et al.*, 2007; Hutchins *et al.*, 2007; Levitan *et al.*, 2007; Kranz *et al.*, 2010; Garcia *et al.*, 2011; Boatman *et al.*, 2017).

We observed that the Chl *a*:C ratio varied 1.7-fold, peaking in the mid-CO_2_ treatment (Table 2). This is in contrast to previous research which showed that both Chl *a*:C and growth rate were largely independent of CO_2_ in *Trichodesmium* (Kranz *et al.*, 2009, 2010). One possible explanation for the difference between our study and previous research is that we grew *Trichodesmium* at a higher light intensity (400 µmol photons m^–2^ s^–1^ in our experiments; 200 µmol photons m^–2^ s^–1^ used by Kranz *et al.*), and that pigment synthesis was down-regulated in our low-CO_2_ treatment where CO_2_ limited growth rate. Down-regulation of pigment synthesis by CO_2_ limitation on growth was also observed previously for *Synechococcus* by Fu *et al.* (2007). In contrast, we hypothesize that the reduction in Chl *a*:C that we observed from mid-CO_2_ to high-CO_2_ may be due to the reduced cost of operating a CCM at high-CO_2_.

### Dark respiration and maintenance metabolic rate

Maintenance metabolism is a collection of key functions necessary to preserve cell viability that are commonly assumed to be independent of growth rate and as such can be estimated by extrapolating the relationship between light-limited growth rate and light intensity (E) to E=0 (Geider and Osborne, 1989). We estimated a maintenance metabolic rate [0.034 d^–1^ ~0.12 mmol O_2_ (g C)^–1^ h^–1^] from previous observations of the light dependence of *Trichodesmium* growth (Boatman *et al.*, 2017; Supplementary Fig. S5). In the dark, *Trichodesmium* has a respiration rate [R_d_=0.60–0.66 mmol O_2_ (g cellular C)^–1^ h^–1^] (Table 3) that is about five times higher than what is needed to maintain cellular metabolism. In most cyanobacteria, dark respiration rates (R_d_) are a small proportion of light-saturated net O_2_ production rates (P_n,max_) (Matthijs and Lubberding, 1988; Geider and Osborne, 1989). In contrast, we observed R_d_ rates that were a high proportion of light-saturated photosynthesis, consistent with other reports of high ratios of R_d_ to net photosynthesis in natural (Kana, 1993) and cultured *Trichodesmium* (Berman-Frank *et al.*, 2001; Kranz *et al.*, 2010; Eichner *et al.*, 2017). For *Trichodesmium* spp., a high R_d_ is required to support the rates of N_2_ fixation measured in darkness. N_2_ fixation is energetically expensive, requiring a minimum consumption of 13 ATP and 6 reducing equivalents per N_2_ fixed, assuming complete recycling of H_2_ produced by nitrogenase to recover ATP.

From our observed growth rates and the C:N ratio reported by Boatman *et al.* (2018*a*), we calculate that the O_2_ consumption rate required to support the level of N_2_ fixation needed for growth is ~0.5 mmol O_2_ (g C)^–1^ h^–1^ in cells grown under low-CO_2_, increasing to ~1 mmol O_2_ (g C)^–1^ h^–1^ in cells grown under high-CO_2_ (Supplementary File SIII). These rates are of the same order as R_d_ [0.60–0.66 mmol O_2_ (g C)^–1^ h^–1^] (Table 3), indicating that R_d_ may provide a substantial proportion of the ATP and reductant required for N_2_ fixation in the light. Significantly, Kana (1993) found that the addition of DCMU to illuminated cells caused the rate of oxygen uptake (U_0_) to decline to the rate observed in darkness, opening up the possibility that continuation of the ‘dark’ respiration rate in the light provides at least some of the energy required for N_2_ fixation.

### Effect of acclimation to pCO_2_ on gross photosynthesis and photosynthetic quotients

Carbon-specific rates are directly related to changes in the specific growth rate, as both rates can be expressed in equivalent units of inverse time (e.g. h^–1^ or d^–1^). However, due to differences in the Chl *a*:C ratio (Table 2), Chl *a*-specific maximum rates (E_0Chl,max_) and initial slopes (α_gChl_) of light-dependent gross photosynthesis did not differ significantly between CO_2_ treatments (Supplementary Fig. S3; Supplementary Table S3), an observation that is consistent with the findings reported by Levitan *et al.* (2007) and Eichner *et al.* (2014). We suggest that the reduced Chl *a*:C ratio at low-CO_2_ relative to mid-CO_2_ is probably due to the cost of up-regulating the CCM, whereas the reduced Chl *a*:C ratio at high-CO_2_ relative to mid-CO_2_ may be due to an increase in carbohydrate storage granules.

In contrast to E_0C,max_, which clearly peaked in the mid-CO_2_ treatment, the maximum net O_2_ evolution rate (P_nC,max_) increased from low- to mid-CO_2_ but was not statistically different between mid- and high-CO_2_ treatments (Table 3). The 2-fold increase of P_nC,max_ from low- to mid- and high-CO_2_ is consistent with the effect of CO_2_ on growth rate.

Dividing the rate of net O_2_ evolution (P_nC_) by the rate of C fixation under comparable light and CO_2_ conditions gave values for the *PQ* that ranged from 1.0 mol O_2_ to 1.3 mol O_2_ evolved per mol CO_2_ fixed (Table 4). A *PQ* of 1.0 is expected if carbohydrate is the major product of photosynthesis as P_nC_ measures the light-driven electron transport from PSII to NADPH, which then feeds into CO_2_ assimilation by the Calvin cycle. A *PQ* >1.0 is expected if recent photosynthate is used in synthesis of compounds that are more reduced than carbohydrates (e.g. lipid) and/or that photosynthetically generated reductant is used to power N_2_ fixation and N assimilation into amino acids. Alternatively or in addition, a slightly higher *PQ* may be required if photosynthetically generated reductant is required for operation of the CCM, or for salvaging CO_2_ that leaks from carboxysomes by conversion of CO_2_ to HCO_3_^–^ by NDH-I_4_ (Price *et al.* (2008). This corroborates our findings, where under high-CO_2_, when the CCM is probably down-regulated, we observed a *PQ* value close to 1.0. Conversely at low- and mid-CO_2_, when more energy is required for the CCM, we observed a *PQ* value >1.0.

### Effect of acclimation to pCO_2_ on light-stimulated O_2_ consumption and the relationship between net and gross O_2_ evolution

We found that O_2_ consumption rates (U_0_) of *Trichodesmium* increased markedly with light intensity, with U_0_ saturating at a similar light intensity to gross O_2_ evolution (Fig. 1). This suggests that light-driven U_0_ increased in parallel with gross O_2_ evolution and that dark respiration continued at similar rates in the light and dark. Previously, Kana (1993) observed a very slight decline in U_0_ between darkness and low-light intensity in natural *Trichodesmium* colonies, followed by parallel increases of U_0_ and O_2_ evolution (E_0_) with increasing light. Kana (1993) attributed the light-stimulated component of U_0_ to the Mehler reaction, as the addition of DCMU to illuminated cells caused U_0_ to decline to the rate observed in darkness.

The slope of the dependence of U_0C_ on E_0C_ decreased with increasing growth CO_2_ from 0.43 in cultures grown under low-CO_2_ to 0.21 in cultures grown under high-CO_2_ (Table 3). Thus, the light-driven component of U_0_ decreased from ~43% of E_0_ in the low-CO_2_ culture to 21% of E_0_ in the high-CO_2_ culture. Pseudocyclic photophosphorylation coupled to the consumption of O_2_ associated with the Mehler reaction can provide ATP that may be used to support N_2_ fixation, CO_2_ fixation, or for operating a CCM (Miller *et al.*, 1988). The linearity between U_0_ and E_0_ suggests that light-dependent O_2_ consumption may be required to balance the ratio of ATP production to NADPH production in the light reactions of photosynthesis. As the photon efficiency of ATP production by pseudocyclic photophosphorylation is the same as that of photophosphorylation driven by linear photosynthetic electron transport (LPET) (Baker *et al.*, 2007), our results suggest that 26% more ATP than can be generated by LPET is required by cells growing under high-CO_2_, increasing to 55% more ATP in cells growing under mid-CO_2_ and 75% more ATP in cells growing under low-CO_2_. Much of the additional ATP that could be generated by pseudocyclic photophosphorylation may be accounted for by the ATP requirements for CO_2_ fixation by the Calvin cycle (1.5 ATP/e^–^) and N_2_ fixation (2.1 ATP/e^–^) being greater than the ratio of ATP to reducing equivalents generated by LPET (1.25 ATP/e^–^). At low-CO_2_, the increase in U_0_ may also be required to operate a CCM, as observed in the freshwater *Synechococcus* (Miller *et al.*, 1988).

### An estimate of the cost of operating the CCM

Stimulation of growth and productivity of *T. erythraeum* IMS101 in response to increasing CO_2_ is commonly attributed to reductions in the amount of energy required for establishing, maintaining, and operating a CCM (Hutchins *et al.*, 2007, 2015; Levitan *et al.*, 2007; Garcia *et al.*, 2011). Two aspects for the cost of operating the CCM in *Trichodesmium* are those for HCO_3_^–^ transport into the cell, which must balance CO_2_ fixation and CO_2_ leakage, and those for retaining inorganic C within the cell by converting CO_2_ that diffuses from the carboxysome to HCO_3_^–^ via the NDH-I_4_ CO_2_ uptake/salvage system. The cost of HCO_3_^–^ transport of ~1 ATP for each C transported into the cell (Raven *et al.*, 2014) could be supplied by either cyclic photosynthetic electron transfer around PSI or pseudocyclic photosynthetic electron transfer linked to the Mehler reaction. As the ratio of CO_2_ leakage to gross inorganic C uptake has been found to be independent of pCO_2_ over the range of 150–1000 µmol mol^–1^ (Kranz *et al.*, 2009, 2010), the ATP required for HCO_3_^–^ transport will also be independent of pCO_2_.

Rather than fuelling ATP production by pseudocyclic electron transport, light-dependent O_2_ consumption may be a consequence of operating the NDH-I_4_ CO_2_ uptake/salvage system. For *Trichodesmium*, the NDH-I_4_ protein is thought to reduce the efflux of CO_2_ from the cell, converting it to HCO_3_^–^ but at a cost of consuming reducing equivalents [NADPH or reduced ferredoxin (F_d_)] (Price *et al.*, 2008).

The stoichiometry based on NADPH as the electron donor can be represented as:

$$2\text{ NADPH}+\;2{\text{ CO}}_{2}+{\text{O}}_{2}\to 2{\text{ HCO}}_{3}^{-}+2{\text{ NADP}}^{+}$$ (9)

If NDH-I_4_ activity is employed to minimize CO_2_ effluxes, then the rate of O_2_ consumption associated with this process would be expected to decrease with increases of extracellular CO_2_ concentration. As NADPH consumption by this mechanism is closely linked in time and space to NADPH production via LPET, this process would be inhibited by DCMU in a similar manner to the Mehler reaction.

### Roles of photosynthetic and respiratory metabolism in N_2_ fixation

Increased pCO_2_ will not only stimulate CO_2_ fixation, but will also stimulate N_2_ fixation (dependent on carbon skeletons for sequestration of the ammonium produced) and growth in *Trichodesmium* (Barcelos e Ramos *et al.*, 2007; Hutchins *et al.*, 2007; Levitan *et al.*, 2007, 2010*b*; Kranz *et al.*, 2009, 2010). This is probably in response to energy relocation from the CCM (Badger *et al.*, 2006; Kranz *et al.*, 2011) toward CO_2_ and N_2_ fixation (Levitan *et al.*, 2007; Kranz *et al.*, 2011).

Diazocytes may use the light reactions of photosynthesis to provide some or most of the ATP required to support N_2_ fixation either through cyclic photophosphorylation associated with electron transfer around PSI or through pseudocyclic photophorylation involing LPET from H_2_O to O_2_ involing both PSII and PSI. However, LPET evolves O_2_, which is known to inactivate nitrogenase. Enhanced rates of the Mehler reaction, unless uncoupled from O_2_ evolution at PSII, will not affect the O_2_ balance of diazocytes. However, if respiration provides the ATP for N_2_ fixation, then sugars and/or organic acids may be produced by photosynthesis in diazocytes prior to the initiation of N_2_ fixation (temporal separation of CO_2_ fixation from N_2_ fixation) or in other cells within the trichome and transported laterally into the diazocytes (spatial separation of CO_2_ fixation from N_2_ fixation).

Temporal separation of N_2_ fixation from photosynthetic O_2_ evolution may be achieved if glycogen that accumulates in diazocytes prior to the onset of N_2_ fixation provides the reducing equivalents and ATP to fuel N_2_ fixation, perhaps supplemented by high rates of cyclic photosynthetic electron transfer around PSI to generate ATP. Temporal separation is consistent with the pattern of CO_2_ fixation and N_2_ fixation observed in *Trichodesmium* where the former peaks earlier in the day than the latter (Berman-Frank *et al.*, 2001). Spatial separation has been observed in heterocystous cyanobacteria where transport of sugars into heterocysts from surrounding cells can be respired to fuel N_2_ fixation (Wolk, 1968; Böhme, 1998). Although we are not aware of direct evidence for rapid transfer of metabolites amongst cells along a trichome, such a transfer would be consistent with observations of Finzi-Hart *et al.* (2009), showing that all cells within a *Trichodesmium* trichome show the same temporal pattern of accumulation and mobilization of cyanophycin granules and the same temporal pattern of labelling with ^13^CO_2_ and ^15^N.

### Conclusion

In this study, we accredit the bell-shaped CO_2_ response of the C-specific maximum gross photosynthesis rates (E_0C,max_) to the CCM, where the 2-fold increase in E_0C,max_ from low- to mid-CO_2_ supports the almost 2-fold increase in balanced growth rates and the decrease in E_0C,max_ from mid- to high-CO_2_ is due to less expenditure on the CCM whilst cells grow at a similar rate.

Our results indicate a significant decrease in the ratio of the rate of light-driven O_2_ consumption to the rate of gross photosynthetic O_2_ evolution with increasing CO_2_, which probably arises from a reduced cost of operating the CCM. In addition, dark respiration appears to be sufficient to provide much of the energy required to support significant rates of N_2_ fixation, even in the light.

We have not extrapolated our findings to a full day as light–response curves were measured at one time point and *Trichodesmium* exhibits pronounced diurnal variability in photosynthetsis and N_2_ fixation (Berman-Frank *et al.*, 2001). In addition, extrapolating to future conditions in the natural environment should consider (i) the impact of adaptation of *Trichodesmium* to future conditions (Hutchins *et al.*, 2015); (ii) strain and clade variability (Hutchins *et al.*, 2013); and (iii) additional integrated effects of abiotic variables other than CO_2_ (i.e. light intensity, temperature, and nutrients such as P and Fe) (Walworth *et al.*, 2016).

In the context of open oceans, nutrient-replete P_n_ and growth rates of *T. erythraeum* IMS101 would have been severely CO_2_ limited at the last glacial maximum relative to current conditions. Future increases of CO_2_ may not significantly increase growth and productivity of *Trichodesmium*, although increases in key stoichiometric ratios (N:P and C:P) as reported by Boatman *et al.* (2018*a*) may affect bacterial and zooplankton metabolism, the pool of bioavailable N, the depth at which sinking organic matter is remineralized, and consequently carbon sequestration via the biological carbon pump (Mulholland *et al.*, 2004; McGillicuddy, 2014). These responses could serve as a negative feedback to climate change by increasing new N and C production, thereby increasing the organic carbon sinking to the deep ocean.

## Supplementary data

Supplementary data are available at *JXB* online.

**Table S1.** The measured and modelled effective light absorption coefficients and relative photosynthetic pigment contribution to the total light absorption.

**Table S2.** The physiological parameters of the N-specific light–response curves for gross and net photosynthetic O_2_ evolution of *T. erythraeum* IMS101.

**Table S3.** The physiological parameters of the Chl *a*-specific light–response curves for gross and net photosynthetic O_2_ evolution of *T. erythraeum* IMS101.

**Table S4.** Values of the goodness of fit for the C-specific light–response curves for gross and net photosynthetic O_2_ evolution of *T. erythraeum* IMS101.

**Table S5.** The Chl *a*-specific photosynthetic quotients of *T. erythraeum* IMS101.

**Fig. S1.** The inorganic carbon chemistry of *T. erythraeum* IMS101 cultures over the light period.

**Fig. S2.** The N-specific light–response curves for gross O_2_ evolution, O_2_ consumption, and net photosynthesis for *T. erythraeum* IMS101.

**Fig. S3.** The Chl *a*-specific light–response curves for gross O_2_ evolution, O_2_ consumption, and net photosynthesis for *T. erythraeum* IMS101.

**Fig. S4.** The measured and modelled *in vivo* light absorption spectra for *T. erythraeum* IMS101.

**Fig. S5.** The light compensation point of *T. erythraeum* IMS101 growth.

**Fig. S6.** The relative emission spectra of the culturing and MIMS LEDs and the spectral corrected light absorption spectra of *T. erythraeum* IMS101.

**File S1.** *In vivo* light absorption.

**File S2.** Modelling the *in vivo* light absorption from pigment absorption spectra.

**File S3.** Stoichiometry and energetics of N_2_ fixation.

## Supplementary Material

Supplementary Material
