# Supplementary material for: CO2 modulation of the rates of photosynthesis and light-dependent O2 consumption in Trichodesmium
Source: J Exp Bot. 2018 Oct 30;70(2):589–97. doi: 10.1093/jxb/ery368 (PMC6322564; doi:10.1093/jxb/ery368)
Supplement: Supplementary Material [file ery368_suppl_supplementary_material.pdf]

Supplementary data

CO_2 modulation of the rates of photosynthesis and light-dependent O_2 consumption in *Trichodesmium*

TOBIAS G. BOATMAN*, PHILLIP A. DAVEY, TRACY LAWSON, RICHARD J. GEIDER

*Address correspondence to t.boatman@imperial.ac.uk

Contents

I. *In vivo* light absorption.

II. Modelling the *in vivo* light absorption from pigment absorption spectra.

III. Stoichiometry and energetics of N_2 fixation.

IV. References.

I. In vivo light absorption.

A 100 ml sample of culture was vacuum-filtered onto a cyclopore 47 mm filter (1-µm pore; Whatman 60750), re-suspended in 10 ml YBCII media and 3 ml pipetted into a quartz cuvette (1 cm pathlength). Triplicate measurements of the spectral light absorption (400 – 800 nm) from each culture were measured using a Hitachi U-3000 spectrophotometer fitted with an integrating sphere (Hitachi High-Technologies, UK). Light absorption spectra were corrected for residual scattering (Suggett *et al.*, 2004) and normalised to total carbon, nitrogen or chlorophyll *a* (Chl) using the C:N and Chl *a*:C ratio of the culture, respectively. The C-specific (m^2 g C^{-1}), wavelength dependent light absorption (a_C (λ)) was calculated using the following equation (Subramaniam *et al.*, 1999);

$$a_C(\lambda) = \left(\frac{OD\ (\lambda) \cdot 2.303 \cdot 100}{[C]}\right) \quad (S1)$$

where C is the total carbon content (g m^3); OD (λ) is the optical density at a specific wavelength (λ = 400 – 700 nm), 2.303 converts from $\log_{10}$ to the natural log base e and 100 from cm^{-1} to m^{-1}.

The effective light absorption coefficient under the culture LED light source was determined according to Morel (1978);

$$a_{C,eff} = \frac{\sum_{\lambda=400}^{700} E(\lambda) \cdot a_C(\lambda)}{\sum_{\lambda=400}^{700} E(\lambda)} \quad (S2)$$

where $a_{C,eff}$ is the C-specific (m^2 g C^{-1}) effective light absorption coefficient (λ = 400 – 700 nm); E (λ) is the relative quanta of the culture LEDs at a specific wavelength (λ = 400 – 700 nm) as determined from the emission spectra (Supplementary Fig. S6); and a_C is the C-specific, wavelength dependent light absorption (m^2 g C^{-1}). Chlorophyll *a*- ($a_{Chl,eff}$ (m^2 g Chl a^{-1})) and N-specific ($a_{N,eff}$ (m^2 g N^{-1})) effective light absorption coefficients were calculated from $a_{C,eff}$ using the Chl *a*:C and C:N ratio of the culture, respectively.

II. Modelling the in vivo light absorption from pigment absorption spectra.

In vivo light absorption spectra were reconstructed using the light absorption spectra of Chl *a* and photoprotectant carotenoids (PPC) taken from Woźniak *et al.* (1999), as well as for phycourobilins (PUB1, PUB2, PUBx, PUB4, PUB5a, PUBb, PUB5d, PUB5g and PUB5j),

phycoerythrins (PE1, PE2a, PE2b and PE3b), alloplastocyanin (APC) and plastocyanins (PC1 and PC2) taken from Küpper *et al.* (2009).

The Chl *a*-specific light absorption coefficient was modelled as the sum of the contributions of all pigments;

$$a_{Chl,mod}(\lambda) = \sum_i \cdot \beta_i \cdot a_i(\lambda) \quad (S3)$$

where $a_{Chl,mod}$ is the modelled *in vivo* light absorption at a specific wavelength (λ = 400 – 700 nm); β^i is the contribution of each pigment to $a_{Chl,mod}$; and a^i is the pigment-specific spectral absorption coefficient of pigment i, in m^2 (g pigment i)$^{-1}$.

A reduced sum of squares method (Sigmaplot 11.0) was used to fit $a_{Chl,mod}$ (λ) to the measured light absorption spectra between 400 and 700 nm. If a zero value was returned for a β^i parameter, that pigment was removed from the model and the curve fit reapplied.

III. Stoichiometry and energetics of N_2 fixation.

N_2 fixation requires a minimum consumption of 16 ATP and 8 reducing equivalents per N_2 fixed:

$$N_2 + 8\,H^+ + 8\,e^- + 16\,ATP \rightarrow 2\,NH_3 + H_2 + 16\,ADP + 16\,Pi \quad (S4)$$

where H^+ is the hydrogen ion and e^- represents a reducing equivalent (Bothe *et al.*, 2010). If the H_2 produced by this reaction is recycled to recover ATP, then the net requirement is 13 ATP and 6 reducing equivalents per N_2 fixed:

$$N_2 + 6\,H^+ + 6\,e^- + 13\,ATP \rightarrow 2\,NH_3 + 13\,ADP + 13\,Pi \quad (S5)$$

In darkness, N_2 fixation must be fuelled by dark respiration. Production of reducing equivalents and ATP via glycolysis and the Krebs cycle can be summarized as:

$$C_6H_{12}O_6 + 6\,H_2O + 4\,ADP + 4\,Pi \rightarrow 6\,CO_2 + 4\,ATP + 24\,e^- + 12\,H^+ \quad (S6)$$

Metabolism of 0.25 molecules of glucose via this mechanism can provide the 6 reducing equivalents (e^-) that are required to fix 1 molecule of N_2 together with 4 ATP. The additional 9 ATP that are required can be provided by metabolism of an additional 0.28 molecules of glucose

via glycolysis and the Krebs cycle when coupled to respiratory electron transport to O_2 coupled to phosphorylation:

$$C_6H_{12}O_6 + 6\ O_2 + 30\ \text{ADP} + 30\ \text{Pi} \rightarrow 6\ CO_2 + 6\ H_2O + 30\ \text{ATP} \quad \text{(S7)}$$

Taking the above into account, and assuming that there are no inefficiencies in the respiratory pathways, the overall stoichiometry of N_2 fixation is approximately:

$$N_2 + 0.61\ C_6H_{12}O_6 + 2.4\ O_2 \rightarrow 2\ NH_3 + 3.7\ CO_2 + 0.61\ H_2O \quad \text{(S8)}$$

Based on the observed growth rates and C:N ratios measured under the same low CO_2 and high CO_2 conditions, respectively (Boatman *et al.*, 2018), and the assumption that N_2 fixation is restricted to 5 hours of illumination, rates of respiratory O_2 consumption necessary to support N_2 fixation can be calculated as:

$$R_d = \left(\frac{1.2\ \text{mol}\ O_2}{\text{mol N}}\right)\cdot\left(\frac{1\ \text{mol N}}{7.9\ \text{mol C}}\right)\cdot\left(\frac{1\ \text{mol C}}{12\ \text{g C}}\right)\cdot\left(\frac{0.2}{\text{d}}\right)\cdot\left(\frac{1\ \text{d}}{5\ \text{h}}\right) = \left(\frac{0.51\ \text{mmol}\ O_2}{\text{g C·h}}\right) \quad \text{(S9)}$$

$$R_d = \left(\frac{1.2\ \text{mol}\ O_2}{\text{mol N}}\right)\cdot\left(\frac{1\ \text{mol N}}{7.3\ \text{mol C}}\right)\cdot\left(\frac{1\ \text{mol C}}{12\ \text{g C}}\right)\cdot\left(\frac{0.36}{\text{d}}\right)\cdot\left(\frac{1\ \text{d}}{5\ \text{h}}\right) = \left(\frac{0.99\ \text{mmol}\ O_2}{\text{g C·h}}\right) \quad \text{(S10)}$$

If the fixed N is incorporated into amino acids, then additional ATP and reducing equivalents would be required. Additional ATP would also be required for protein synthesis or accumulation of the N-rich reserve polymer cyanophycin (Allen and Weathers, 1980).

IV. References.

Allen MM, Weathers PJ. 1980. Structure and composition of cyanophycin granules in the cyanobacterium *Aphanocapsa* 6308. *Journal of bacteriology* **141**, 959-962.

Boatman TG, Lawson T, Geider RJ. 2017. A Key Marine Diazotroph in a Changing Ocean: The Interacting Effects of Temperature, CO_2 and Light on the Growth of *Trichodesmium erythraeum* IMS101. *PLoS One* **12**, e0168796.

Boatman TG, Mangan NM, Lawson T, Geider RJ. 2018. Inorganic carbon and pH dependency of photosynthetic rates in *Trichodesmium*. *Journal of Experimental Botany*, ery141-ery141.

Bothe H, Schmitz O, Yates MG, Newton WE. 2010. Nitrogen fixation and hydrogen metabolism in cyanobacteria. *Microbiology and molecular biology reviews* **74**, 529-551.

Eichner M, Thoms S, Kranz SA, Rost B. 2015. Cellular inorganic carbon fluxes in *Trichodesmium*: a combined approach using measurements and modelling. *Journal of Experimental Botany* **66**, 749-759.

Küpper H, Andresen E, Wiegert S, Šimek M, Leitenmaier B, Šetlík I. 2009. Reversible coupling of individual phycobiliprotein isoforms during state transitions in the cyanobacterium *Trichodesmium* analysed by single-cell fluorescence kinetic measurements. *Biochimica et Biophysica Acta (BBA)-Bioenergetics* **1787**, 155-167.

Morel A. 1978. Available, usable, and stored radiant energy in relation to marine photosynthesis. *Deep Sea Research* **25**, 673-688.

Raven JA, Beardall J, Giordano M. 2014. Energy costs of carbon dioxide concentrating mechanisms in aquatic organisms. *Photosynthesis research* **121**, 111-124.

Subramaniam A, Carpenter EJ, Karentz D, Falkowski PG. 1999. Bio-optical properties of the marine diazotrophic cyanobacteria *Trichodesmium* spp. I. Absorption and photosynthetic action spectra. *Limnology and Oceanography* **44**, 608-617.

Suggett DJ, MacIntyre HL, Geider RJ. 2004. Evaluation of biophysical and optical determinations of light absorption by photosystem II in phytoplankton. *Limnology and Oceanography: Methods* **2**, 316-332.

Woźniak B, Dera J, Ficek D, Majchrowski R, Kaczmarek S, Ostrowska M, Koblentz-Mishke O. 1999. Modelling the influence of acclimation on the absorption properties of marine phytoplankton. *Oceanologia*, 187-210.

Table S1. *The mean (± S.E.) measured and modelled effective light absorption coefficients and relative contribution of each photosynthetic pigment to the total light absorption under the culturing LEDs within* T. erythraeum IMS101, *when acclimated to three target CO_2 concentrations (Low = 180 µmol mol^{-1}, Mid = 380 µmol mol^{-1} and High = 720 µmol mol^{-1}), saturating light intensity (400 µmol photons m^{-2} s^{-1}) and optimal temperature (26 °C).*

**Variables**	**Units**	**Low CO_2**		**Mid CO_2**		**High CO_2**	
$a_{Chl,eff}$	m^2 (g Chl *a*)$^{-1}$	8.8 (0.8)		8.0 (0.5)		7.0 (0.5)	
$a_{C,eff}$	m^2 (g C)$^{-1}$	0.034 (0.003)	[A]	0.062 (0.003)	[B]	0.038 (0.003)	[A]
$a_{N,eff}$	m^2 (g N)$^{-1}$	0.232 (0.021)	[A]	0.424 (0.020)	[B]	0.258 (0.018)	[A]
$a_{Chl,mod}$	m^2 (g Chl *a*)$^{-1}$	8.8 (0.8)		8.1 (0.5)		7.1 (0.5)	
$a_{C,mod}$	m^2 (g C)$^{-1}$	0.034 (0.003)	[A]	0.063 (0.003)	[B]	0.039 (0.003)	[A]
$a_{N,mod}$	m^2 (g N)$^{-1}$	0.233 (0.020)	[A]	0.431 (0.020)	[B]	0.263 (0.018)	[A]
Chl *a*	%	35.82 (2.81)		38.74 (2.02)		42.82 (1.10)	
PPC	%	30.72 (2.24)		29.79 (2.66)		26.29 (0.44)	
PUB1	%	1.27 (1.27)	[A]	4.73 (2.10)		7.29 (1.85)	[B]
PUB2	%	2.18 (0.62)		0.94 (0.62)		0.75 (0.75)	
PE1	%	10.22 (2.61)		9.15 (2.23)		4.12 (1.28)	
PE2a	%	1.93 (1.93)		0		0	
PE3b	%	9.17 (1.32)		10.13 (2.69)		11.48 (0.38)	
APC	%	4.83 (1.85)		5.46 (2.69)		6.88 (0.79)	
PC2	%	3.87 (1.31)	[B]	1.06 (1.05)		0.35 (0.21)	[A]

Light absorption coefficients were corrected to the culture LEDs and were normalised to a chlorophyll *a* (m^2 g Chl *a*$^{-1}$) carbon (m^2 g C^{-1}) and nitrogen (m^2 g N^{-1}) basis. Abbreviations; $a_{Chl,eff}$, $a_{C,eff}$ and $a_{N,eff}$ are the effective Chl *a*-, C- and N-specific light absorption coefficients, while $a_{Chl,mod}$, $a_{C,mod}$ and $a_{N,mod}$ are the modelled Chl *a*-, C- and N-specific light absorption coefficients. Light absorption spectra were reconstructed from a range of pigment absorption spectrums ($\lambda = 400 – 700$); comprising chlorophyll *a* (Chl *a*), photoprotectant carotenoid (PPC), phycourobilins (PUB1, PUB2), phycoerythrin (PE1, PE2a and PE3b), alloplastocyanin (APC) and plastocyanin (PC2). Letters in parenthesis indicate significant differences between CO_2 treatments (One Way ANOVA, Tukey post hoc test; $P < 0.05$); where [B] is significantly greater than [A].

Table S2. *The physiological parameters (± S.E.) of the N-specific light-response curves for the gross and net photosynthetic O_2 evolution of* T. erythraeum IMS101 *(n = 4) measured using the MIMS light source.*

**Parameters**	**Units**	**Low CO_2**		**Mid CO_2**		**High CO_2**	
Gross O_2 evolution							
$E_{0N,max}$	mmol O_2 (g N)$^{-1}$ h^{-1}	12.76 (0.81)		25.39 (1.17)		18.62 (0.99)	
E_k	µmol photons m^{-2} s^{-1}	277 (15)		250 (20)		281 (15)	
α_{gN}	µmol O_2 (g N)$^{-1}$ h^{-1} (µmol photons m^{-2} s^{-1})$^{-1}$	0.046 (0.002)		0.103 (0.005)		0.066 (0.001)	
Net Photosynthesis							
$P_{nN,max}$	mmol O_2 (g N)$^{-1}$ h^{-1}	7.40 (0.14)	[A]	16.95 (1.92)	[A]	14.48 (0.87)	[B]
E_k	µmol photons m^{-2} s^{-1}	300 (41)		270 (24)		270 (10)	
α_{nN}	µmol O_2 (g N)$^{-1}$ h^{-1} (µmol photons m^{-2} s^{-1})$^{-1}$	0.026 (0.003)	[A]	0.063 (0.007)	[A]	0.054 (0.002)	[B]
R_{dN}	mmol O_2 (g N)$^{-1}$ h^{-1}	-4.085 (0.533)	[A]	-4.311 (0.881)	[B]	-4.124 (0.082)	

Abbreviations; $E_{0N,max}$, the N-specific maximum gross O_2 evolution rate; $P_{nN,max}$, the N-specific maximum net O_2 evolution rate; E_k, the light saturation parameter; α_{gN} and α_{nN} are the N-specific initial slopes the light response curve for net and gross photosynthesis; R_{dN}, the N-specific dark respiration rate. Letters in parenthesis indicate significant differences between CO_2 treatments (One Way ANOVA, Tukey post hoc test; $P < 0.05$); where [B] is significantly greater than [A].

Table S3. *The physiological parameters (± S.E.) of the Chl a-specific light-response curves for the gross and net photosynthetic O_2 evolution of* T. erythraeum IMS101 *(n = 4) measured using the MIMS light source.*

**Parameters**	**Units**	**Low CO_2**		**Mid CO_2**		**High CO_2**	
Gross O_2 evolution							
$E_{0Chl,max}$	mol O_2 (g Chl *a*)$^{-1}$ h^{-1}	0.481 (0.026)		0.483 (0.005)		0.546 (0.025)	
E_k	µmol photons m^{-2} s^{-1}	277 (15)		250 (20)		281 (15)	
α_{gChl}	mmol O_2 (g Chl *a*)$^{-1}$ h^{-1} (µmol photons m^{-2} s^{-1})$^{-1}$	1.75 (0.15)		1.97 (0.16)		1.95 (0.03)	
Net Photosynthesis							
$P_{nChl,max}$	mol O_2 (g Chl *a*)$^{-1}$ h^{-1}	0.280 (0.015)	[A]	0.320 (0.022)	[A]	0.425 (0.023)	[B]
E_k	µmol photons m^{-2} s^{-1}	300 (41)		270 (24)		270 (10)	
α_{nChl}	mmol O_2 (g Chl *a*)$^{-1}$ h^{-1} (µmol photons m^{-2} s^{-1})$^{-1}$	0.97 (0.11)	[A]	1.20 (0.11)	[A]	1.58 (0.06)	[B]
R_{dChl}	mol O_2 (g Chl *a*)$^{-1}$ h^{-1}	-0.152 (0.014)	[A]	-0.081 (0.014)	[B]	-0.121 (0.002)	

Abbreviations; $E_{0Chl,max}$, the Chl *a*-specific maximum gross O_2 evolution rate; $P_{nChl,max}$, the Chl *a*-specific maximum net O_2 evolution rate; E_k, the light saturation parameter; α_{gChl} and α_{nChl} are the Chl *a*-specific initial slopes the light response curve for net and gross photosynthesis; R_{dChl}, the Chl *a*-specific dark respiration rate. Letters in parenthesis indicate significant differences between CO_2 treatments (One Way ANOVA, Tukey post hoc test; $P < 0.05$); where [B] is significantly greater than [A].

Table S4. *Values of the goodness of fit for the C-specific light-response curves for the gross and net photosynthetic O_2 evolution.*

**Curve fits**	**Low CO_2**				**Mid CO_2**				**High CO_2**			
	Rep 1	Rep 2	Rep 3	Rep 4	Rep 1	Rep 2	Rep 3	Rep 4	Rep 1	Rep 2	Rep 3	Rep 4
Gross O_2 evolution												
n	26	26	24	27	27	28	27	27	27	26	26	26
Parameters	2	2	2	2	2	2	2	2	2	2	2	2
r^2	0.983	0.988	0.991	0.994	0.986	0.997	0.997	0.997	0.996	0.988	0.992	0.996
Adjusted r^2	0.982	0.988	0.990	0.993	0.985	0.997	0.996	0.997	0.996	0.988	0.992	0.996
p-value	<.0001	<.0001	<.0001	<.0001	<.0001	<.0001	<.0001	<.0001	<.0001	<.0001	<.0001	<.0001
Net O_2 evolution												
n	26	26	24	27	27	28	27	27	27	26	26	26
Parameters	3	3	3	3	3	3	3	3	3	3	3	3
r^2	0.973	0..917	0.960	0.982	0.979	0.997	0.979	0.987	0.993	0.991	0.974	0.993
Adjusted r^2	0.970	0.910	0.956	0.981	0.977	0.997	0.978	0.986	0.993	0.991	0.972	0.992
p-value	<.0001	<.0001	<.0001	<.0001	<.0001	<.0001	<.0001	<.0001	<.0001	<.0001	<.0001	<.0001

Table S5. *The photosynthetic quotients (± S.E.) for* T. erythraeum IMS101*, calculated from the light saturated, maximal rates of Chl a-specific O_2 evolution and the C-fixation rates.*

**Parameters**	**Units**	**Low CO_2**	**Mid CO_2**	**High CO_2**
Photosynthetic quotient				
E_{0Chl}/V_{Chl}	mol O_2 (mol C)$^{-1}$	2.20 (0.19)	1.90 (0.07)	1.36 (0.04)
P_{nChl}/V_{Chl}	mol O_2 (mol C)$^{-1}$	1.24 (0.11)	1.21 (0.06)	1.07 (0.05)

V_{Chl} was calculated as $V_{Chl} = (V_{Chl,max} \cdot [CO_2])/(K_m + [CO_2])$ using the value of K_m from Boatman *et al.* (2018) and $[CO_2]$ from Table 1. E_{0Chl} was calculated as $E_{0Chl} = E_{0Chl,max} \cdot (1 - e(-\alpha \cdot E/E_{0Chl,max})$ and P_{nChl} was calculated as $P_{nChl} = P_{nChl,max} \cdot (1 - e(-\alpha \cdot E/P_{nChl,max})$ using E = 400 µmol photons m^{-2} s^{-1} and values of E_k from Supplementary Table S4.

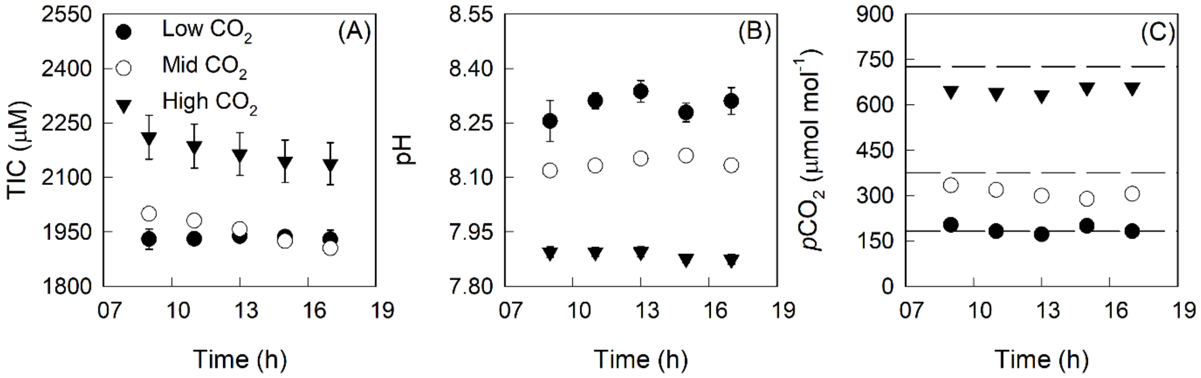

Fig. S1. The inorganic carbon chemistry (Ci) of *T. erythraeum* IMS101 cultures, measured at two-hour intervals over the light period. Filtered samples were taken to measure the total inorganic CO_2 (TIC) (A) and $pH_{(Total)}$ (B). The CO_2 drift (C) was calculated via *CO2SYS* using the measured pH and TIC, with the same constants as reported in Boatman *et al.* (2017).

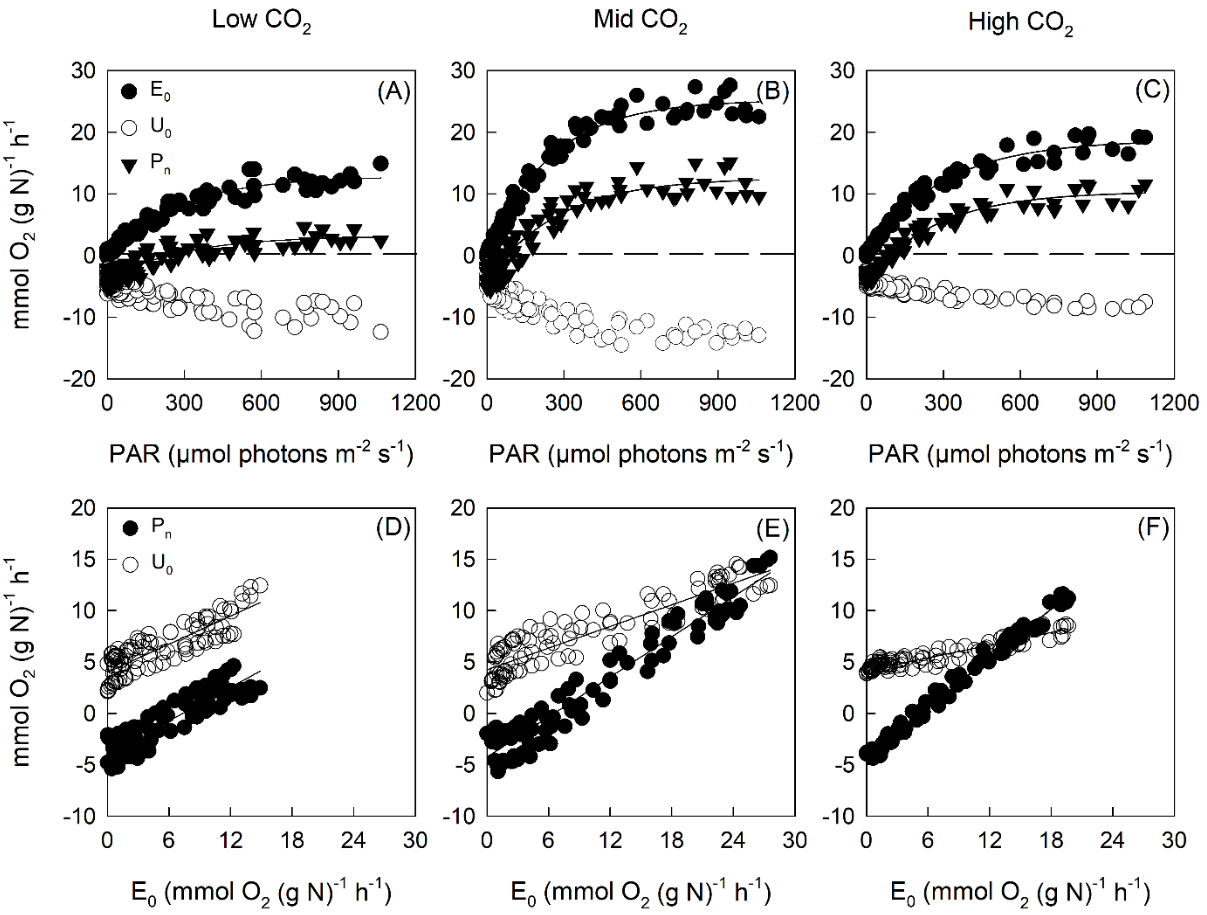

Fig. S2. The N-specific light response curves for gross O_2 evolution, O_2 consumption and net photosynthesis (n = 4) (A-C) and the relationship between both gross O_2 evolution and net O_2 evolution or O_2 consumption (D-F) for *T. erythraeum* IMS101. Oxygen evolution rates are normalised to a chlorophyll *a* basis (mmol O_2 (g N)$^{-1}$ h^{-1}). The dashed line represents where gross O_2 evolution equals O_2 consumption (i.e. net photosynthesis = 0).

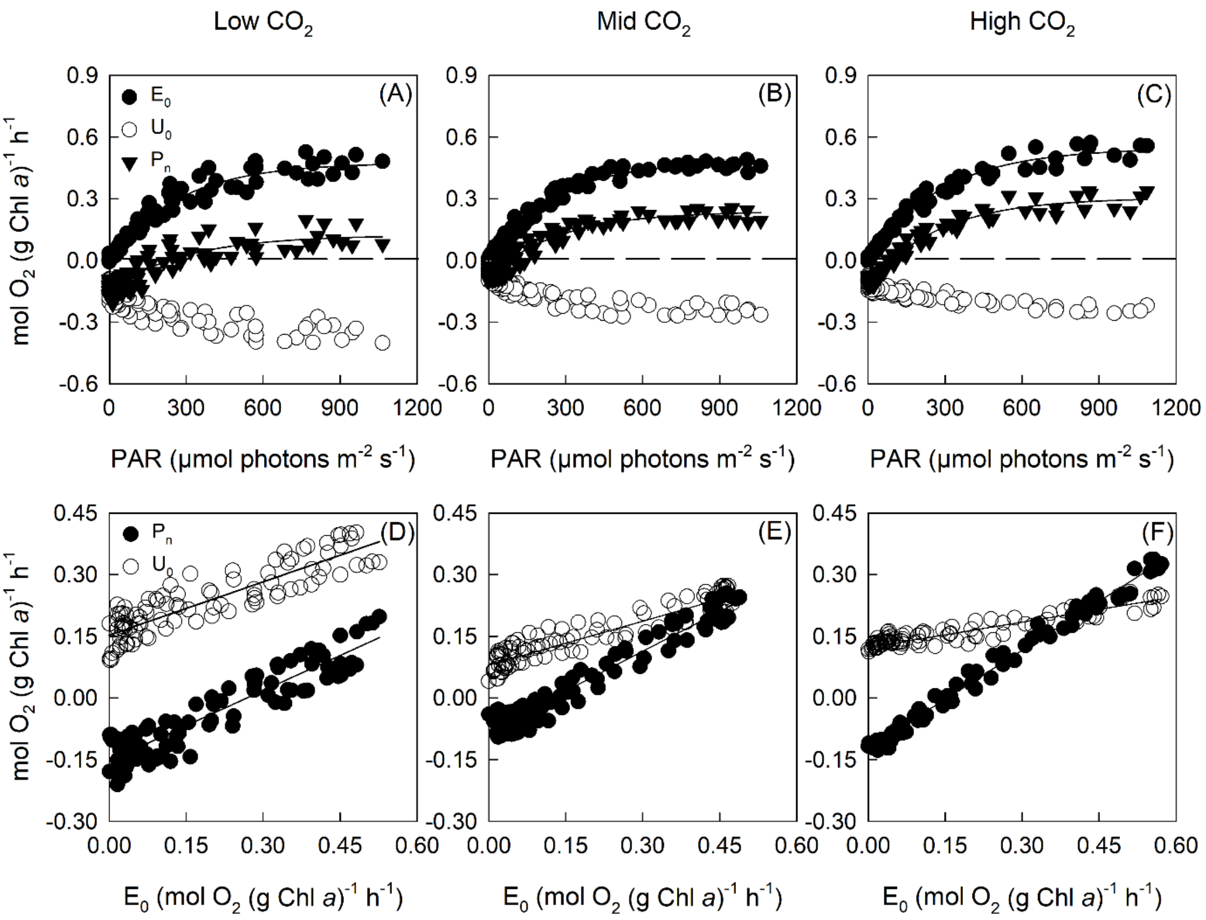

Fig. S3. The Chl *a*-specific light response curves for gross O_2 evolution, O_2 consumption and net photosynthesis (*n* = 4) (A-C) and the relationship between gross O_2 evolution and net O_2 evolution or O_2 consumption (D-F) for *T. erythraeum* IMS101. Oxygen evolution rates are normalised to a chlorophyll *a* basis (mol O_2 (g Chl *a*)$^{-1}$ h^{-1}). The dashed line represents where gross O_2 evolution equals O_2 consumption (i.e. net photosynthesis = 0).

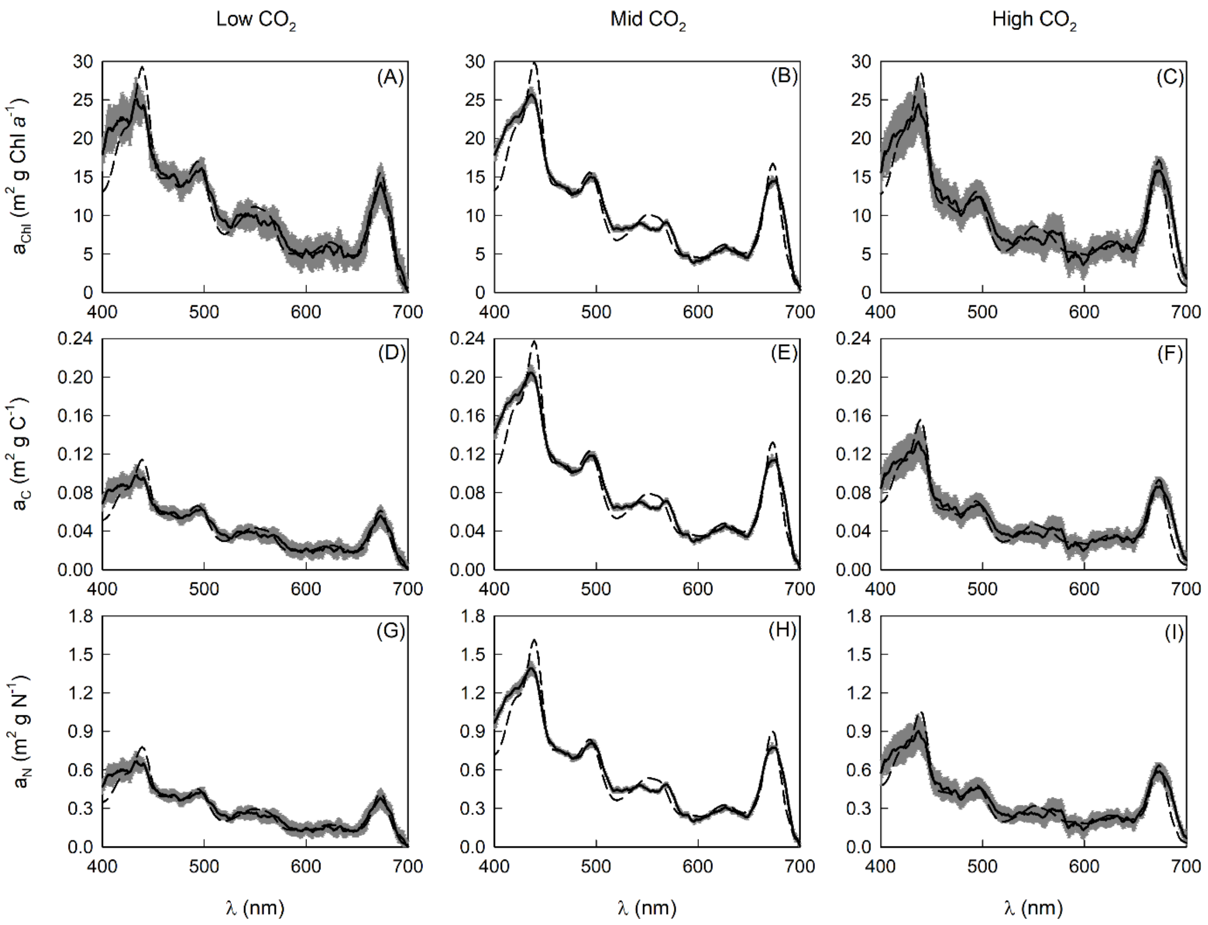

Fig. S4. The mean (± S.E.) Chl *a*- (A-C), C- (D-F) and N-specific (G-I) *in vivo* light absorption spectra for *T. erythraeum* IMS101 ($n = 4$). Cultures were acclimated to three CO_2 concentrations (Low = 180 µmol mol^{-1}, Mid = 380 µmol mol^{-1} and High = 720 µmol mol^{-1}), saturating light intensity (400 µmol photons m^{-2} s^{-1}) and optimal temperature (26 °C). The solid black line is the measured effective light absorption spectra (grey area represents the S.E.) while the dashed line is the modelled effective light absorption spectra.

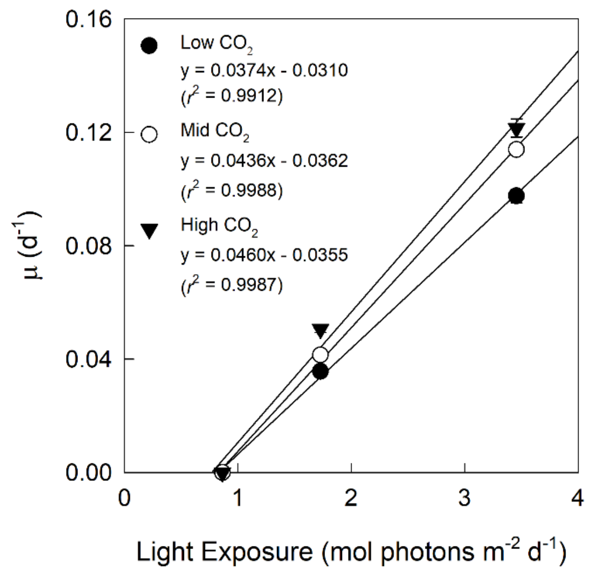

Fig. S5. The relationship between light-limited growth rate and light exposure (LE) for *T. erythraeum* IMS101 (Data taken from Boatman *et al.* (2017)). The intercept at LE = 0 provides the estimate of the maintenance metabolic rate of about 0.034 d^{-1} = 0.0014 h^{-1}, which assuming a respiratory quotient of 1 O_2/CO_2, equates to a maintenance respiration rate of 0.12 mmol O_2 (g C)$^{-1}$ h^{-1}.

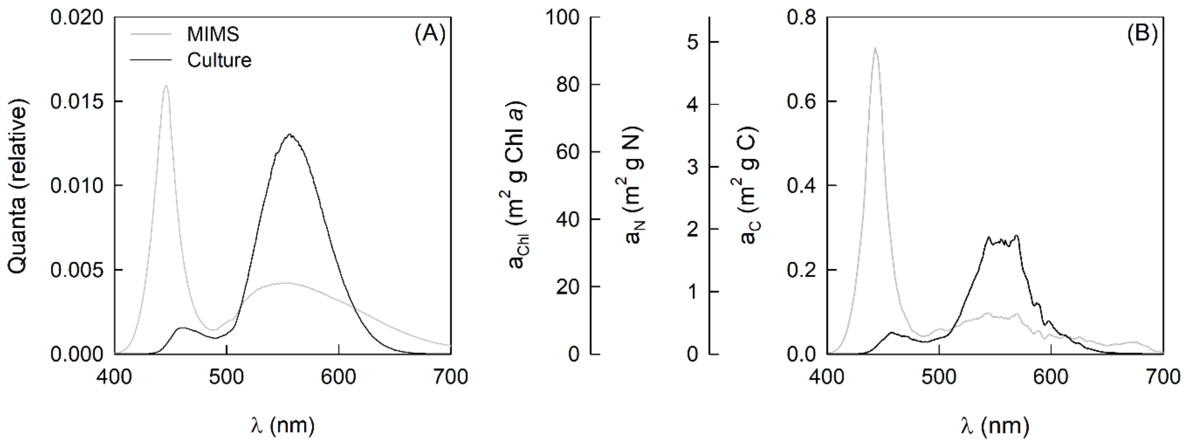

Fig. S6. The relative emission spectra of the white Iso Light 400 LED block used for O_2 evolution MIMS incubations (Grey line) and the culturing LEDs (Black line) (A). An example of the Chl *a*-, N- and C-specific *in vivo* light absorption spectra for *T. erythraeum* IMS101 (380 µmol mol^{-1} CO_2 treatment), corrected to the MIMS and Culture LEDs (B).
